# Factors associated with successful publication for systematic review protocol registration: an analysis of 397 registered protocols

**DOI:** 10.1186/s13643-023-02210-8

**Published:** 2023-06-02

**Authors:** Le Huu Nhat Minh, Huu-Hoai Le, Gehad Mohamed Tawfik, Omar Mohamed Makram, Thuan Tieu, Luu Lam Thang Tai, Dang The Hung, Van Phu Tran, Karim Mohamed Shahin, Ali Ahmed-Fouad Abozaid, Jaffer Shah, Nguyen Hai Nam, Nguyen Tien Huy

**Affiliations:** 1 International Ph.D. Program in Medicine, College of Medicine, Taipei Medical University, 110 Taipei, Taiwan; 2 Global Clinical Scholars Research Training Program, Harvard Medical School, Boston, MA USA; 3 Research Center for Artificial Intelligence in Medicine, Taipei Medical University, Taipei, Taiwan; 4 Faculty of Medicine, University of Medicine and Pharmacy at Ho Chi Minh City, Ho Chi Minh City, 700000 Vietnam; 5 Online Research Club (https://onlineresearchclub.org/), Nagasaki, 852-8523 Japan; 6 Faculty of Medicine, Ain Shams University, Cairo, Egypt; 7 Faculty of Medicine, October 6 University, Giza, Egypt; 8 McMaster University, Hamilton, Ontario L8S 4L8 Canada; 9 Department of Emergency, City Children’s Hospital, Ho Chi Minh, Vietnam; 10 Tra Vinh University, Tra Vinh City, Vietnam; 11 Faculty of Medicine, Alexandria University, Alexandria, Egypt; 12 Weill Cornell Medicine, New York, NY USA; 13 Department of Liver Tumor, Cancer Center, Cho Ray Hospital, Ho Chi Minh City, Vietnam; 14 School of Tropical Medicine and Global Health, Nagasaki University, 1-12-4 Sakamoto, Nagasaki, 852-8523 Japan

**Keywords:** Protocol, Systematic review, Meta-analysis, Protocol registration, PROSPERO, Cochrane, JBI

## Abstract

**Background:**

Meta-analyses are on top of the evidence-based medicine pyramid, yet many of them are not completed after they are begun. Many factors impacting the publication of meta-analysis works have been discussed, and their association with publication likelihood has been investigated. These factors include the type of systematic review, journal metrics, h-index of the corresponding author, country of the corresponding author, funding sources, and duration of publication. In our current review, we aim to investigate these various factors and their impact on the likelihood of publication. A comprehensive review of 397 registered protocols retrieved from five databases was performed to investigate the different factors that might affect the likelihood of publication. These factors include the type of systematic review, journal metrics, h-index of the corresponding author, country of the corresponding author, funding sources, and duration of publication.

**Results:**

We found that corresponding authors in developed countries and English-speaking countries had higher likelihoods of publication: 206/320 (*p* = 0.018) and 158/236 (*p* = 0.006), respectively. Factors affecting publications are the countries of corresponding author (*p* = 0.033), whether they are from developed countries (*OR*: 1.9, 95% *CI*: 1.2–3.1, *p* = 0.016), from English-speaking countries (*OR*: 1.8, 95% *CI*: 1.2–2.7, *p* = 0.005), update status of the protocol (*OR*: 1.6, 95% *CI*: 1.0–2.6, *p* = 0.033), and external funding (*OR*: 1.7, 95% *CI*: 1.1–2.7, *p* = 0.025). Multivariable regression retains three variables as significant predictors for the publication of a systematic review: whether it is the corresponding author from developed countries (*p* = 0.013), update status of the protocol (*p* = 0.014), and external funding (*p* = 0.047).

**Conclusion:**

Being on top of the evidence hierarchy, systematic review and meta-analysis are the keys to informed clinical decision-making. Updating protocol status and external funding are significant influences on their publications. More attentions should be paid to the methodological quality of this type of publication.

**Supplementary Information:**

The online version contains supplementary material available at 10.1186/s13643-023-02210-8.

## Introduction

Systematic reviews (SR) and meta-analyses (MA) are considered to be the highest tier of the evidence-based medicine pyramid due to their ability to arrive at an empirical algorithm for diseases by combining results from different studies conducted over many years [1]. However, several studies point out that many registered SRs are not published for a long time after registration. For example, about 20% of Cochrane protocols were not published as full reviews within 8 years [2, 3], while another study reported that 26% of PROSPERO protocols (from February 2011 to February 2012) remained unpublished after at least 65 months [4].

Recently, some publications have shed light on the topic of registered protocols that were not published. One study related the lack of publication to financial factors, finding that funded reviews were more likely to be published [4, 5]. A survey in 2009, in which 625 authors participated, found that lack of time, funding, and organizational support were the main barriers to finishing the reviews [6]. Lack of time was also cited by a survey in 2018, which concluded that SRs with protocols took more than twice the time from search to submission than SRs without protocols [2], while other studies need even more time — up to 2.4 years [3].

Nevertheless, these publications were limited in scope, and the set of factors that may impact the likelihood of publication of registered protocols is not well understood. Studies may restrict their attention to reviews registered in one database, such as PROSPERO, and miss other databases such as Cochrane, Joanna Briggs Institute (JBI), Campbell, and others.

In our current review, we investigated various factors that might affect the likelihood of publication. These points included the different registration databases, types of SR, the h-index of the corresponding author, publication date, duration of study work time, funding sources, and journal metrics at the time of publication.

## Methods

### Identifying protocols: search terms and inclusion/exclusion criteria

**Fig. 1:**
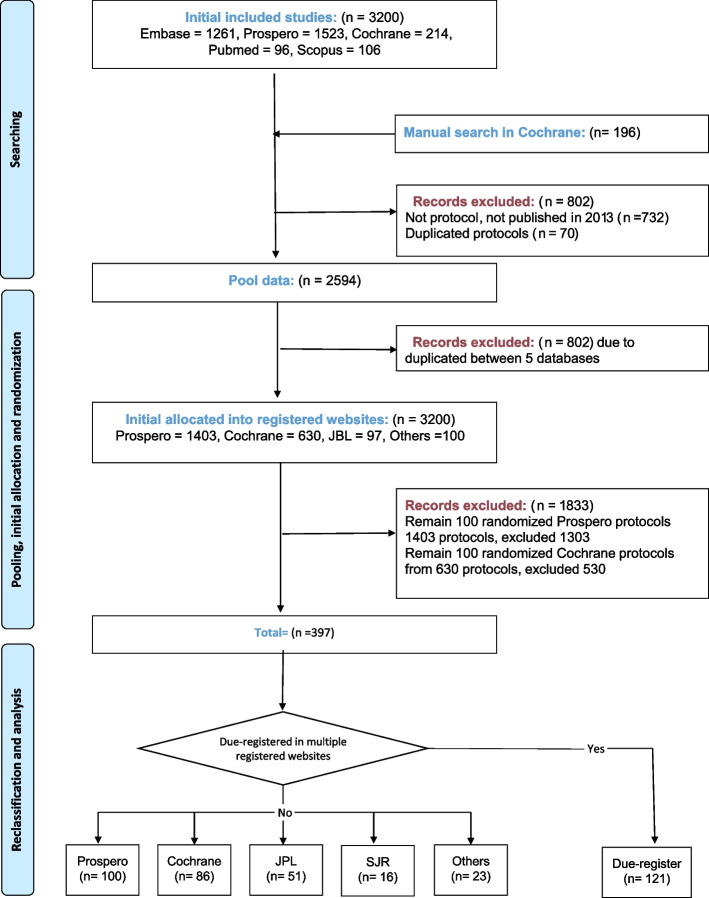
Flow diagram for study scheme steps

Five databases (Embase, PROSPERO, Cochrane Database of Systematic Reviews, PubMed, and Scopus) were searched for SR/MA protocols published in 2013 with the following terms in their title: protocol, systematic review, and meta-analysis. We selected this period because it represented the maximal time that a protocol would have the chance to be published (5 years) from the year in which we conducted this search method (26 July 2018), as estimated from the previous study [7]. After searching, two independent authors examined the results and excluded papers that either were abstract only, not SR/MA protocols, or had no available full text. Any discrepancies were resolved by discussion and consensus among the senior author (N. T. H). The protocols were later divided into five groups according to their registered databases: PROSPERO, Cochrane, JBI, SJR, and others. The other databases include the *Annals of Cardiothoracic Surgery*, *BMC Medical Research Methodology*, *BMC Psychiatry*, *Clinical & Translational Allergy*, *BMJ Open*, *Environmental Evidence*, *Implementation Science*, *Injury Prevention*, *International Journal of Medical Informatics*, *International Journal of Stroke*, *Journal of Agricultural and Food Chemistry*, *Journal of Medical Internet Research, Primary Care, Respiratory Journa*l, *Research Journal of Applied Sciences*, *Engineering and Technology*, *BMC Trials*, and *World Journal of Surgical Oncology*. For groups with more than 100 protocols, 100 were randomly chosen for analysis using random function in Excel. For groups with less than 100 protocols, all the protocols were examined. Figure 1 outlines the selection process.

#### Definition of variables

A summary of the variables’ definition and reasons for collecting could be found in Supplemental Table 1.

#### Published and unpublished protocols

We recorded various factors that might be associated with an increased likelihood of publication, as described below. Published protocols are protocols that were used in preparing at least one publication. Unpublished protocols either (a) produced no publications or (b) had been registered for use in at least one publication, but the publication in question was withdrawn. Except where noted, this information came from the published protocol.

To check whether the project was published after protocol registration, we employed the following steps. Firstly, the protocol was searched for the authors’ indication that the project was published. If there was not a statement, the protocol’s title, the first author’s name, and then the protocol’s registered number were searched on Google, Google Scholar, and PubMed. Finally, we looked for the first author, corresponding author, protocol title, and registered numbers on ResearchGate and checked if the protocol was published under another name.

#### Scopus’s h-index of corresponding author

The h-index is an indication of the researchers, which would increase the likelihood that it would be published. We collected information on the h-index of the corresponding authors of each protocol. The h-index was retrieved from Scopus (Elsevier, Amsterdam, Netherlands) between July and December 2018. We used Scopus because of its efficient author identification algorithm [8]. If there was more than one profile for the corresponding author, we selected the one with the higher h-index. We classified the authors into three groups based on the h-index: < 6, 6 < × < 12, and > 12 [9].

#### Country of the corresponding author

Nationality and language are known to be obstacles to publication [10, 11]. Based on the corresponding author of the protocol, we used the Institute for Scientific Information (ISI) list of developed countries to determine whether the author was based in a developing or a developed country [12], as it is known that developing countries usually have lower scientific production due to their financial burden. We also determined whether the corresponding author was based in a native-English-speaking country, defined as Australia, Canada, New Zealand, the UK, or the USA. In our study, the countries of corresponding authors were working countries, not home countries.

#### Corresponding author is the first author

Another variable — “Corresponding author is the first author” — was included, and it was considered positive if the first author and the corresponding author of the article were the same. Someone who is both the corresponding author and the first author may be under additional pressure, which might increase the paper’s chances of publication.

#### Number of authors registered in protocols

The numbers of authors registered in the protocol were also collected, as more authors might impact the length needed to finished a systematic review [7].

#### External co-workers

Because of its nature as a literature reviews, it is easy for systematic reviews to have multinational authorships. External co-workers from another nation might affect the chance of publications considering the geographical and cultural disparities.

#### Registered database

Based on registered databases, the papers were classified into 6 groups: Cochrane only, PROSPERO only, JBI only, SJR only, and duo register (registered in 2 of mentioned databases), and other. Different registers have different standards, which improve the quality and affect the chance to publish [13, 14].

#### Study funding

Funding is a well-known factor affecting publications [15, 16]. Data regarding funding support were collected according to the Cochrane Handbook 5. Supports were classified as “internal” when they are given by the organizations where the review was conducted and “external” when they are supported by other institutions or funding agencies [1, 16, 17].

#### Update status of protocol

The status whether a protocol was updated or not was collected in the registries. As new evidence emerges, changing the protocol would allow research to be up to date and, therefore, increase the chance of publication [3, 18].

#### Type of systematic reviews

Different types of systematic reviews might use different methodologies, which might result in different challenges and publication chances. We adopted a recent classification of systematic reviews, published in 2018, in order to adequately categorize various reviews’ protocols [19]. The systematic reviews were classified as effectiveness reviews, experiential (qualitative) reviews, costs/economic evaluation reviews, prevalence and/or incidence reviews, diagnostic test accuracy reviews, etiology and/or risk reviews, expert opinion/policy reviews, psychometric reviews, prognostic reviews, and methodological systematic reviews.

#### Journal metrics

Elsevier’s Scopus has two older citation analysis metrics: Source Normalized Impact per Paper (SNIP) and SCImago Journal Rank (SJR) [20]. CiteScore is a new metric of Scopus [21]. The factors recorded were the impact factor (IF), the number of citations, CiteScore, SNIP, and SJR.

We used the journal metrics of the review’s publication year. We performed our metrics search between July and December 2018. The Clarivate databases were used to identify the IF, while the Scopus database was used for CiteScore, SNIP, and SJR. For each database, these statistics were described in box plots.

#### Time from protocol registration to paper publication

The publication date of each protocol was retrieved from its respective registered database. If a protocol was co-registered in multiple databases, or there were multiple publications resulted from a protocol, we use the date from the earliest versions. The time from protocol registration to paper publication was calculated from the publication date of the protocol to the publication date of the paper and visualized through box plots for each database.

#### Statistical analysis

Statistical analysis was performed in SPSS statistics version 25. Descriptive analysis was applied to summary protocol characteristics. We used the Mann-Whitney *U*-test and the Kruskal Wallis H-test to determine the difference of continuous factors between groups, while Fisher’s exact test was performed to compare the dichotomous variables (yes or no variables). All factors associated with our outcome of interest, i.e., factors that directly related to the cause of un-publishing protocols in univariable analysis, would be included and re-analyzed in a multivariable logistic regression model. A stepwise approach was perform to determine what variables would enter the regression model. A two-sided *p*-value of < 0.05 was considered statistically significant.

## Results

A total of 3200 protocols were obtained after searching five databases — Embase, PROSPERO, Cochrane, PubMed, and Scopus — along with 196 protocols from Cochrane by manual searching. After applying inclusion/exclusion criteria, 2230 protocols entered the randomization selection stage, which resulted in a final dataset of 397 protocols. One-hundred twenty-one protocols were registered in multiple databases. The number of single-database-registered protocols (i.e., published in one single database) in PROSPERO, Cochrane, and JBI was respectively 100, 86, and 51, and the remaining protocols were allocated in SJR databases (*n* = 16) or other databases (*n* = 23). For further analysis, all of the 397 protocols were later reclassified, as described in Fig. 1.

Regarding protocol characteristics, a majority of corresponding authors were working in developed countries (80.6%) and native-English-speaking countries (59.7%). The median value of the Scopus h-index was 8 with IQR of 14 (range 0–94). There were 301 authors (75.8%) who held both the first author position and the corresponding author position. Apart from corresponding author information, the median number of authors per protocol was 5 with IQR of 3 (range 3–6), and there were about 114 protocols (28.7%) where participation from external co-workers was recognized. A total of 180 protocols (45.4%) declared no funding support. We also found that 118 (29.7%) protocols were updated in the study process after registration. Other information was summarized in Table 1. We also found that protocols which were registered in the JBI database were more likely to be published in journals with a low CiteScore, SNIP score, SJR score, and impact factor (Supplementary Figs. 2, 3, 4 and 5). However, there was no difference in publication chance among databases registered (*p* = 0.186). Approaching author information through the h-index of a corresponding author seems to be unrelated to the publication chance (*p* = 0.118). Protocols with corresponding authors working in developed countries (*p* = 0.019) and native-English-speaking countries (*p* = 0.006) were more likely to be published as papers. Besides that, study funding status (*p* = 0.029), along with an updated status of the protocol (*p* = 0.033), was also recorded as having a relationship with the outcome of interest. The overview information of other factors was presented in Table 1.

**Table 1:** Protocol characteristics comparison between published protocols and unpublished protocols (*N* = 397)

**Protocol’s characteristics**	**Total** **(** ***N*** ** = 397)**	**Published protocols** **(** ***N*** ** = 244)**	**Unpublished protocols** **(** ***N*** ** = 153)**	***p*** **-value**
Information of corresponding author				
Scopus’s h-index of corresponding author				0.118c
Value	8 (14.5)	9.5 (15.5)	5 (3.0)	
Corresponding author based in developed countries				**0.019a**
Yes	320 (80.6)	206 (84.4)	114 (74.5)	
No	77 (19.4)	38 (15.6)	39 (25.5)	
Corresponding author based in English-speaking countries				0.006a
Yes	237 (59.7)	159 (65.2)	78 (51.0)	
No	160 (40.3)	85 (34.8)	75 (49.0)	
Corresponding author is the first author				0.278a
Yes	301 (75.8)	180 (73.8)	121 (79.1)	
No	96 (24.2)	64 (26.2)	32 (20.9)	
Country of corresponding authors				**0.033**b
UK	79 (19.9)	58 (23.8)	21 (13.7)	
Australia	62 (15.6)	44 (18.0)	18 (11.8)	
Canada	46 (11.6)	28 (11.5)	18 (11.8)	
USA	47 (11.8)	26 (10.7)	21 (13.7)	
China	24 (6.0)	14 (5.7)	10 (6.5)	
Others	139 (35.0)	74 (30.3)	65 (42.5)	
Other characteristics				
Number of authors registered in protocols				0.333c
Value	5 (3)	5 (3)	4 (3)	
External co-worker				0.909a
Yes	114 (28.7)	71 (29.1)	43 (28.1)	
No	283 (71.3)	173 (70.9)	110 (71.9)	
Registered database				0.186b
Cochrane only	86 (21.7)	49 (20.1)	37 (24.2)	
PROSPERO only	100 (25.2)	72 (29.5)	28 (18.3)	
JBI only	51 (12.8)	28 (11.5)	23 (15.0)	
SJR	16 (4.0)	10 (4.1)	6 (3.9)	
Others	23 (5.8)	12 (4.9)	11 (7.2)	
Duo register (^a^)	121 (30.5)	73 (29.9)	48 (31.4)	
Study funding				**0.029**b
No funding support	180 (45.4)	104 (42.6)	76 (49.7)	
Internal fund only	74 (18.6)	40 (16.4)	34 (22.2)	
Having external fund (with/without internal fund)	143 (36.0)	100 (41.0)	43 (28.1)	
Protocol was updated				**0.033**a
Yes	118 (29.7)	82 (33.6)	36 (23.5)	
No	279 (70.3)	162 (66.4)	117 (76.5)	
Type of systematic review				0.667b
Effectiveness	311 (78.3)	191 (78.3)	120 (78.4)	
Experiential or qualitative	22 (5.5)	12 (4.9)	10 (6.5)	
Etiology or risk	21 (5.3)	11 (4.5)	10 (6.5)	
Diagnostic test accuracy	16 (4.0)	11 (4.5)	5 (3.3)	
Others	27 (6.8)	19 (7.8)	8 (5.2)	

Statistical analysis tests: a, Fisher’s exact test. b, chi-square test. c, Mann-Whitney *U*-test.

Descriptive information was reported as a, N (%) and b, median (IQR).

^a^One-hundred twenty-one duo-registered protocols were all registered in PROSPERO, along with another database: Cochrane (14), JBI (46), SJR (46), others (15)

All potential protocol characteristics related to being published as papers, determined by univariable analysis, were summarized in Table 2. The multivariable logistic regression shows that whether the corresponding author from developed countries [*OR* = 1.6, 95% *CI* = 1.1–2.6, *p* = 0.013] and updated status of protocols [*OR* = 1.8, 95% *CI* = 1.1–3.0, *p* = 0.014), along with having external funding support *OR* = 1.6, 95% *CI* = 1.0–2.6, *p* = 0.047], were associated with the publication of protocols. Notably, although being recorded as a potential factor, the country of corresponding authors showed no relationship at all with the outcome of interest.

**Table 2:** Factors associated with the paper publication of protocols

**Protocol’s characteristics**	**Univariable analysis**		**Multivariable analysis**	
	**COR (95% ** ***CI*** **)**	***p*** **-value**	**AOR (95% ** ***CI*** **)**	***p*** **-value**
Corresponding author based in developed countries				
Yes	1.9 (1.2–3.1)	**0.016**	1.6 (1.1–2.6)	0.013
No	Reference	-	Reference	**-**
Corresponding author based in English-speaking countries				
Yes	1.8 (1.2–2.7)	**0.005**	**-**	**-**
No	Reference	**-**	-	**-**
Country of corresponding authors				
UK	2.4 (1.3–4.4)	**0.004**	-	-
Australia	2.1 (1.1–4.1)	**0.020**	-	-
Canada	1.4 (0.7–2.7)	0.368	-	-
USA	1.1 (0.6–2.1)	0.805	-	-
China	1.2 (0.5–3.0)	0.644	-	-
Others	Reference	**-**	-	-
Study funding				
Internal fund only	0.9 (0.5–1.5)	0.586	0.7 (0.4–1.4)	0.363
Having external fund (with/without internal fund)	1.7 (1.1–2.7)	**0.025**	1.6 (1.0–2.6)	**0.047**
No funding support	Reference	-	Reference	-
Protocol was updated				
Yes	1.6 (1.0–2.6)	**0.033**	1.8 (1.1–3.0)	**0.014**
No	Reference	-	Reference	-

Statistical analysis tests: logistic regression. Stepwise method was used to select the variables in the multivariable regression. “Corresponding author based in English-speaking countries” and “country of corresponding authors” were insignificant factors in the multivariable regressions and were removed from the model

## Discussion

In this review, we investigated the main factors that made systemic review and meta-analysis protocols more likely to be published. Firstly, we found that corresponding authors in both developed countries (206/320, *p* = 0.018) and English-speaking countries (158/236, *p* = 0.006) had a higher chance of publishing the paper of their registered protocols. We found that the highest percentage of corresponding authors for both protocols and reviews was from the UK, representing 19.9% and 23.8% of the authors, respectively. Our models of multivariable logistic regression revealed that three main factors significantly impact the publication outcome, which included the fact that it is whether the corresponding author from developed countries (*OR* = 1.6, 95% *CI* = 1.1–2.6, *p* = 0.013), the updated protocol status of the published review paper (*OR* = 1.8, 95% *CI*: 1.1–3.0, *p* = 0.014), and external funding (*OR* = 1.6, 95% *CI*: 1.0–2.6, *p* = 0.044) (Table 1). This is most likely explained by more funding opportunities, better background research support, and the fact that English is the primary spoken language in these countries. However, our multivariable analysis did not show any significant difference among these countries compared to the other ones. It should also be noted that most reviews are published in English or included English studies. These results are supported by other studies that found that about 30% of corresponding authors of protocols registered in PROSPERO are from the UK [2, 5].

Contrastingly, another study published in 2016 found that UK corresponding authors were only 16%, while those of China were 21%. These results were based on searching the MEDLINE database [22]. Interestingly, China is demonstrating a rapid growth in the number of meta-analyses conducted, despite being neither a developed nor an English-speaking country [23].

We found that protocols with external funding had a higher chance of being published than those with internal funding. This result is consistent with that of Tsujimoto et al. (2017), who reported that PROSPERO protocols that received funding were associated with better publishing chances [4]. Lack of funding is also considered a barrier to publishing SRs [6]. It even impact researchers’ motivation to do research [16]. However, in terms of quality, it was noticed that reviews funded by internal sources, such as academic institutions, were of a higher quality than those with external funding or those that failed to report funding status [17]. In conclusion, the author should pay attention to funding when conducted the research. Being funded by prestigious funds from major universities or countries comes with the responsibility of fully completing the research and publishing its results. Even though it increased the chance of publication, funding could cause bias to the researchers. Therefore, funds that pose no bias to the results should be prioritized.

It is believed that systematic reviews need to be kept up to date [24]. In 2016, a study revealed that about 10% of published reviews are updated, and remarkably, 81% of these updates were just Cochrane SRs [22]. Another result from Oral Health Cochrane Systematic Reviews showed that 14.5% of all reviews have been updated [25]. Univariate analysis reveals that there is a significant correlation between updated protocol and publication (*OR* = 1.6, 95% *CI* 1.0–2.60, *p* = 0.033), and that 33.6% of the published reviews had updated protocols.

Updated protocols were associated with a change in the author list, but not associated with the time from protocol registration to publication (Supplementary Fig. 1). Previously, it was found that a shorter time to publication might result in higher chances of the review being updated, and that a longer time to publication is often associated with the review having two published protocols, which hints at changes in the review plan [3].

In this study, we noticed that only 61.46% of protocols resulted in publications. In other words, more than one-third of the registered protocols either did not publish their results or are not yet finished. A recent study in 2018 found that about one-third of protocols did not have any publication within 3–5 years; however, only 80 papers were analyzed in the study, which is a relatively small sample size [2]. Two other studies found that this rate of nonpublication reached 12.4%, measured through a survey among investigators, and a second study found that 26% of PROSPERO protocols did not have any publication after 65 months of protocol registration [4, 6]. In 2008, a study found that 19.1% of Cochrane protocols were unpublished [3].

In our study, Cochrane protocols had one of the lowest rates of publication at 57%. This could be explained by the meticulous process that any Cochrane protocol or review must go through, and the editorial process strategies employed by Cochrane that promote good reporting, such as the MECIR standards [26]. JBI protocols had the lowest rate of publication, at only 55%. Many previous studies reported that Cochrane reviews had higher quality reporting methods than non-Cochrane reviews, which might take more time and reduce the overall chance of publication [22, 27]. A recent review suggests that the overall quality of registered reviews is higher than that of non-registered reviews [13].

In our analysis, we found that the median number of authors in a review was 5. A previous study estimated that at least 5 reviewers at an average of 67 weeks are required to complete a well-conducted systematic review [5]. Page et al. (2016) also found that the median number of authors is 5 (*IQR* 4–6) [22]. Two other studies have found that the number of authors is 7 (*IQR* 5–11) and 6. However, both of them had some limitations, such as a small sample size or including protocols from only one database [2, 28].

It has been reported that the best methodological quality SR/MAs were conducted by groups of authors with high levels of scientific experience, with a median h-index of 14 [29]. In 2018, Schreiber et al. found that higher academic rankings among academic physicians in different specialties were associated with higher h-indices. On average, assistant professors have an h-index of 2–5, associate professors 6–10, and full professors 12–24 [9]. In our study, we found that the median h-index of corresponding authors in protocols with published reviews was 9.5, while in unpublished protocols it was only 5. Since JBI is a nursing protocol database, it was associated with the lowest median h-index among corresponding authors.

We adopted a recent classification of systematic reviews, published in 2018, in order to adequately categorize various reviews’ protocols [19]. Interestingly, we discovered that most of the published protocols belonged to the effectiveness group (78.3%). This result was supported by another study published in 2016, which found that about 55% of the SRs were classified as therapeutic, 25% as epidemiology, 11% as diagnosis/prognosis, and 10% as other [22]. Also, when reviews from the Cochrane databases were compared to ones from high-impact journals in cancer, it was found that Cochrane reviews were less likely to address questions concerning prognosis [30].

Remarkably, we found that 30.5% of the protocols in our random sample pool were registered in two protocol databases. Previous studies have found a higher proportion of co-registration of protocols. A total of 45% of non-Cochrane protocols were published in both journals and PROSPERO [31]. A total of 89.2% of protocols published in “BMC *Systematic Reviews*” were also registered in PROSPERO [28].

In boxplot-based analyses of several different scoring systems, we observed considerable impact factor-like potential variability of publications between databases studied. The median scores of dual-published papers did not have substantial variation from the broader sample medians, indicating that dual publication is not likely to influence the usage of a given article. However, individual databases such as Cochrane and JBI often had substantial and potentially significant deviations from the overall means. While a small overall sample size may contribute to some of Cochrane’s deviation, caution should be taken in selecting the database one uses to generate and obtain meta-analyses due to potential bias in article quality and relevance to current topical discourse. Moreover, we found that 14.8% of the Cochrane protocols were withdrawn, compared to the previously reported rate of 12.7% [3]. In the past decade, new emerging journals have made it easier for authors to publish their SRs, even if there is already an SR on the same topic published at the same time. This has resulted in a large number of overlapping studies, and a previous study has estimated that about 67% of meta-analyses have at least one overlapping meta-analysis within 3 years [32].

There were some limitations to our study. We did not cover all the registered SR protocols in 2013. However, we performed a randomization of 100 Cochrane and PROSPERO protocols. Other studies have included more variables like page count, certificate of insurance (COI), funding by the pharmaceutical industry, funding by academic institutions, randomized clinical studies (RCTs) as primary studies, meta-analysis included, journal bibliometrics, author bibliometrics, and others [17], but these were not available in PROSPERO records, so we could not adjust for them in our analysis. The main reasons reported for non-publication were lack of time, overly broad SR scope, and few studies eligible for SRs, as well as rejection [6]. The authors of unpublished protocol were not contacted to confirm whether they stopped the project or not. Only English databases were searched; therefore, the factors affecting the publications of protocols registered in regional databases might differ.

## Conclusion

Systematic review and meta-analysis studies require significant attention and a careful literature review before conducting them. Factors that increase chances of publication include a sufficient timeline, an adequate number of qualified authors, and a good source of external or internal funding. Choosing the most applicable database and periodically updating the review status are highly recommended to ensure a chance of publication among the highest CiteScore journals, since this type of publication is placed on top of the hierarchy of evidence-based medicine and greatly influences clinical practice.

## Supplementary Information

**Additional file 1: Supplementary Fig. 1.** Description of time from protocol registration to paper publication.

**Additional file 2: Supplementary Fig. 2.** Description of Citescore among databases.

**Additional file 3: Supplementary Fig. 3.** Description of SNIP score among databases.

**Additional file 4: Supplementary Fig. 4.** Description of SJR score among databases.

**Additional file 5: Supplementary Fig. 5.** Description of journal’s impact factor among databases.

**Additional file 6: Supplemental Table 1.** Summary of definition and reasonings for collecting for variables.

## Data Availability

All data generated or analyzed during this study are included in this published article and its supplementary information files.
