# Supplementary figures and images for: Factors associated with successful publication for systematic review protocol registration: an analysis of 397 registered protocols
Source: Syst Rev. 2023 Jun 2;12:93. doi: 10.1186/s13643-023-02210-8 (PMC10239197; doi:10.1186/s13643-023-02210-8)

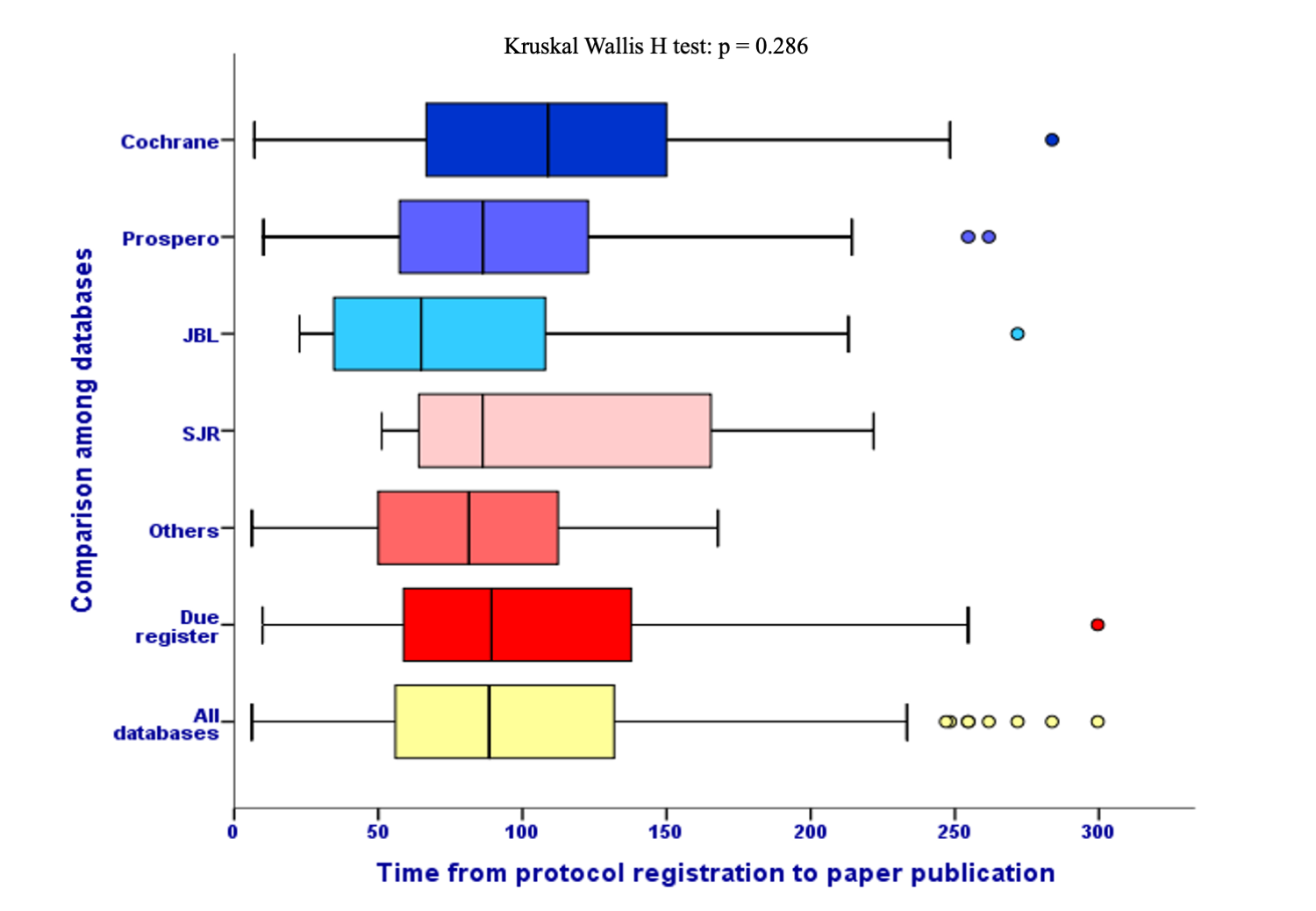

Supplement: Supplementary file 1 — Additional file 1: Supplementary Fig. 1. Description of time from protocol registration to paper publication. [file 13643_2023_2210_MOESM1_ESM.tiff]

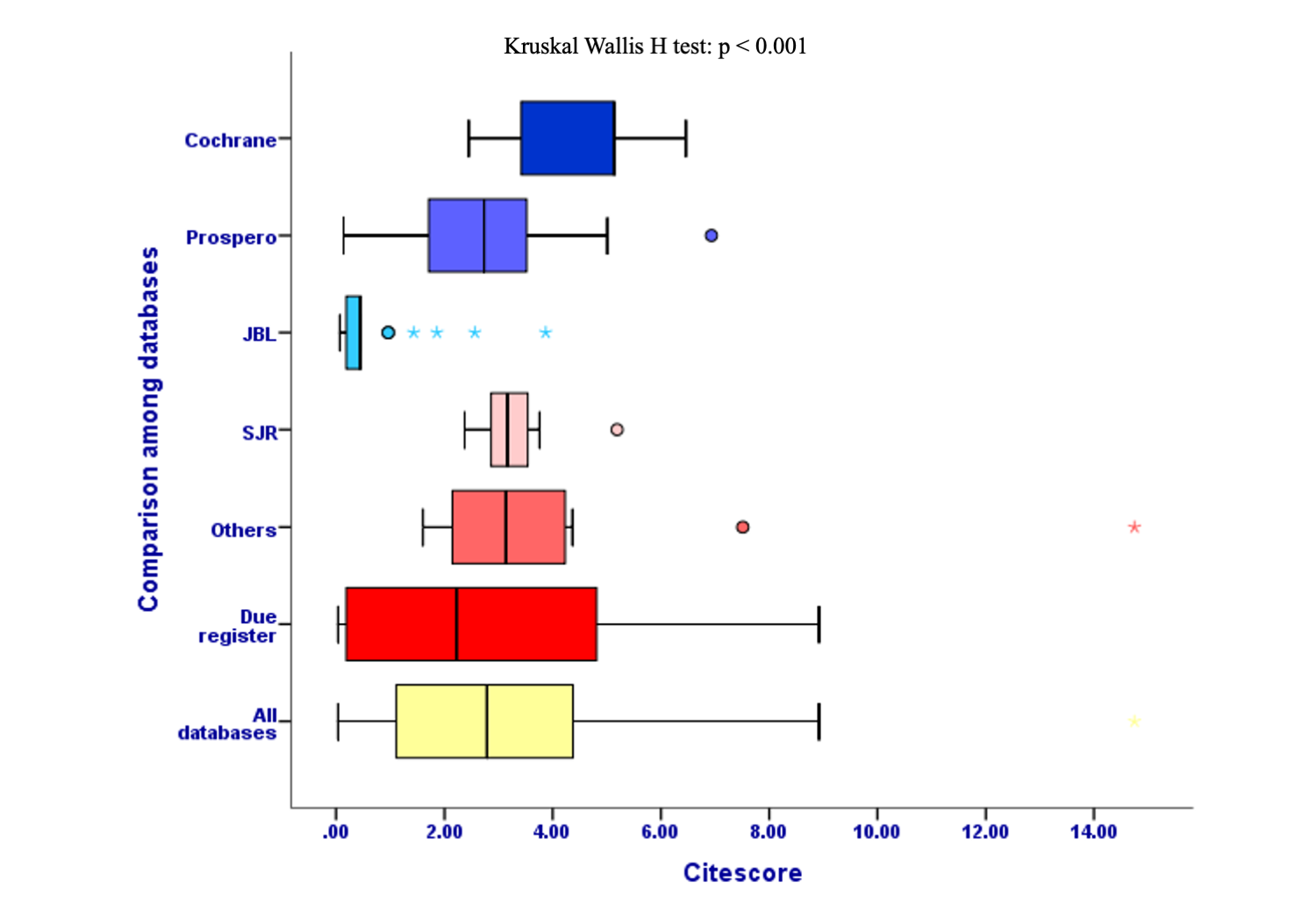

Supplement: Supplementary file 2 — Additional file 2: Supplementary Fig. 2. Description of Citescore among databases. [file 13643_2023_2210_MOESM2_ESM.tiff]

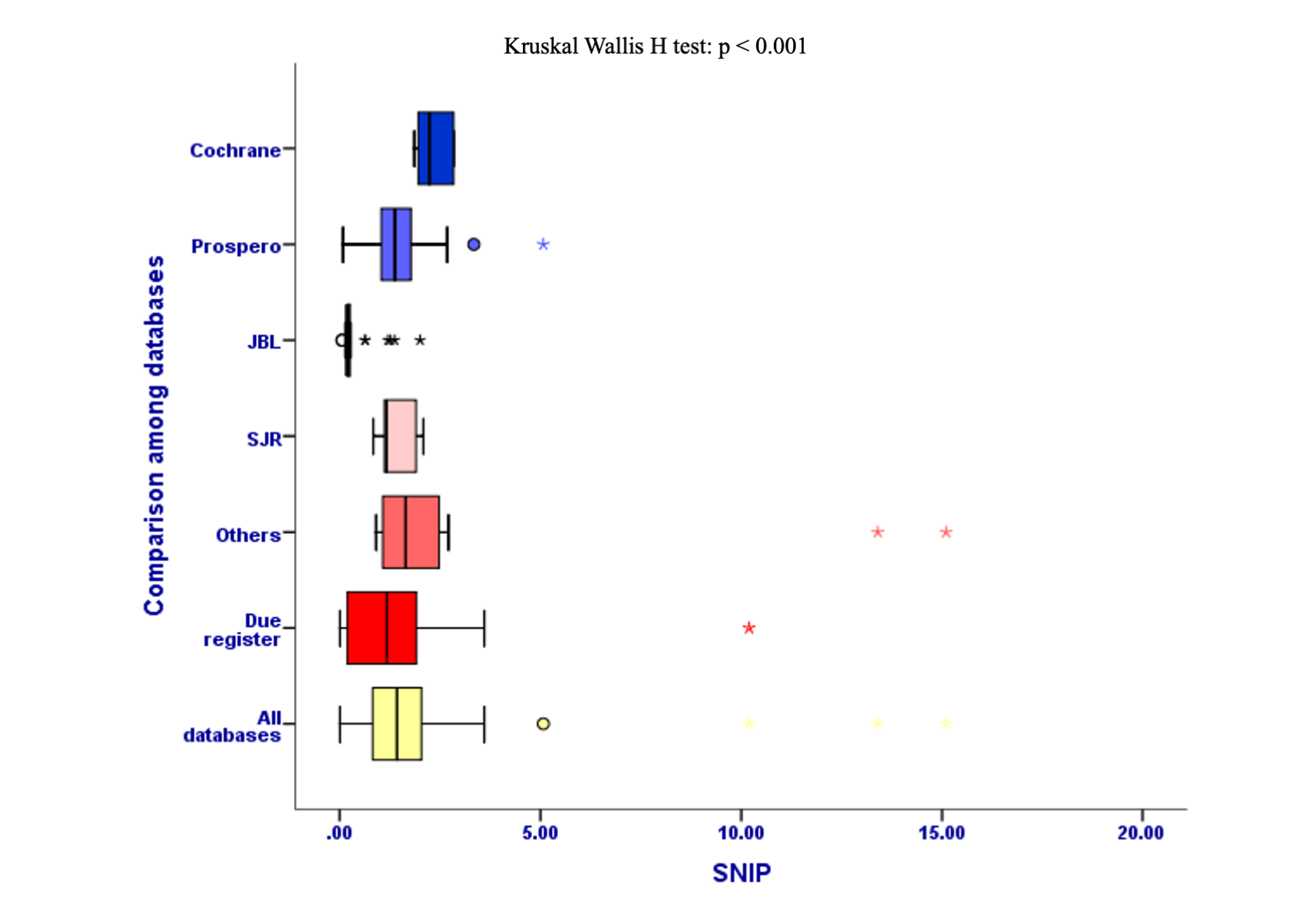

Supplement: Supplementary file 3 — Additional file 3: Supplementary Fig. 3. Description of SNIP score among databases. [file 13643_2023_2210_MOESM3_ESM.tiff]

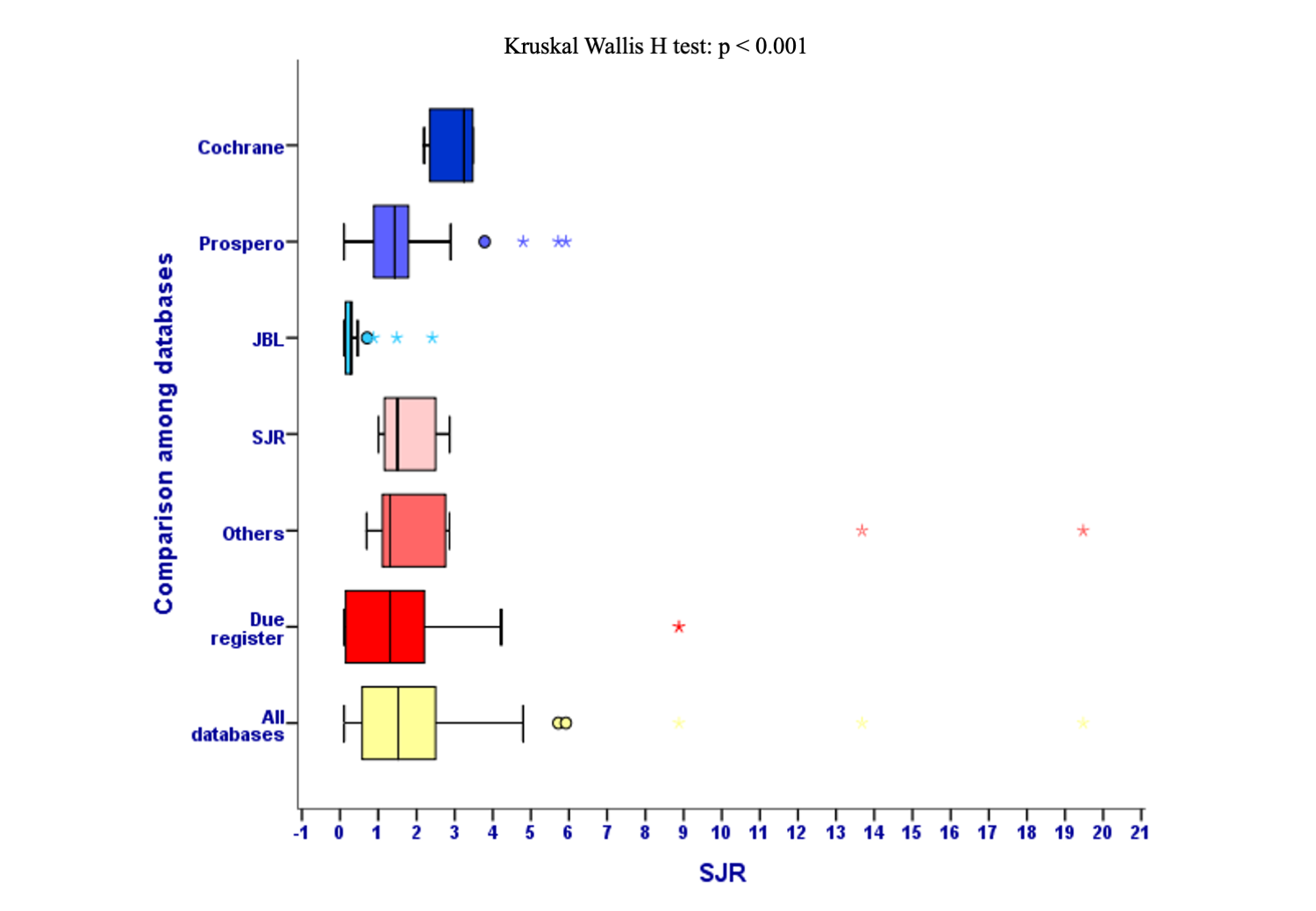

Supplement: Supplementary file 4 — Additional file 4: Supplementary Fig. 4. Description of SJR score among databases. [file 13643_2023_2210_MOESM4_ESM.tiff]

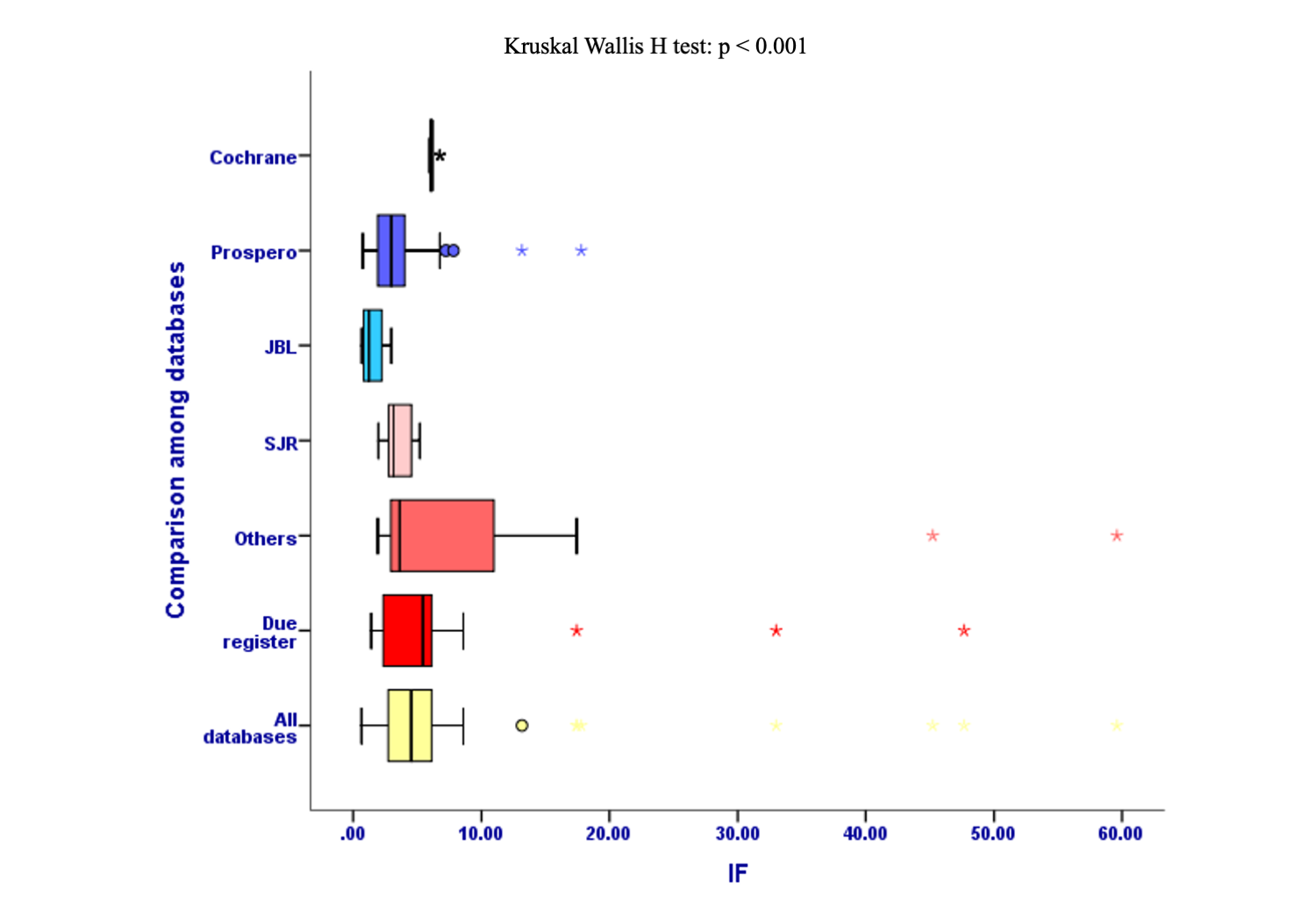

Supplement: Supplementary file 5 — Additional file 5: Supplementary Fig. 5. Description of journal’s impact factor among databases. [file 13643_2023_2210_MOESM5_ESM.tiff]
