# Supplementary material for: Factors associated with successful publication for systematic review protocol registration: an analysis of 397 registered protocols
Source: Syst Rev. 2023 Jun 2;12:93. doi: 10.1186/s13643-023-02210-8 (PMC10239197; doi:10.1186/s13643-023-02210-8)
Supplement: Supplementary file 6 — Additional file 6: Supplemental Table 1. Summary of definition and reasonings for collecting for variables. [file 13643_2023_2210_MOESM6_ESM.docx]

**Supplemental Table 1: Summary of definition and reasonings for collecting for variables.**

| **Variable** | **Definitions** | **Reasons** | **References** |
| --- | --- | --- | --- |
| Published and unpublished protocols | Published protocols are those that resulted in at least one publication. Unpublished protocols either a) didn't correspond with any publications or b) have relate publications that were retracted | This variable is the outcome of concern used in univariable and multivariable analysis. |  |
| Scopus’s h-index of corresponding author | The h-index was acquired from Scopus (Elsevier, Amsterdam, Netherlands) between July and December 2018, and classified into three categories: 6, 6<x<12, and >12 | Higher h-index might be associated with higher quality and therefore higher chances of publication | (Schreiber and Giustini, 2019, Sanz-Cabanillas et al., 2017) |
| Country of the corresponding author | Institute for Scientific Information (ISI) list of developed countries were used to classify the countries into developed or developing. Whether the corresponding author was based in a native English-speaking country was also collected | Countries and languages are known barrier to publication | (Di Bitetti and Ferreras, 2017, Yousefi-Nooraie et al., 2006) |
| Corresponding author is the first author | The condition whether first author and the corresponding author of the article were the same | A person being both corresponding author and first author might have more pressure which potentially lead to higher chances of publication |  |
| Number of authors registered in protocols | Number of authors in protocols | Higher number of members might lead to higher chances of publication | (Borah et al., 2017) |
| External co-worker | Ex-ternal researcher from a different country | It might impact the likelihood of publishing when taking into account geographical and cultural differences |  |
| Registered database | The publications were divided into six groups based on the registered databases: Cochrane only, PROSPERO only, JBI only, SJR only, dual register (registered in two of the listed databases), and other | Registrations increase the quality. Standards might differ between registries. As a result, enrolling in some registries can increase the chance of publication | (Sideri et al., 2018, Petticrew et al., 2002) |
| Study funding | Data on financial assistance were gathered in accordance with Cochrane Handbook 5. Funding were categorized as "external" when provided by institutions or funding organizations other than the organizations where the review was done and "internal" when provided by those organizations. | Funding affects different aspect of research and, therefore, affects publication chances | (Gómez-García et al., 2017, D'Arrietta et al., 2022, Higgins and (editors), 2011) |
| Protocol was updated | Whether a protocol has been updated | Updating the protocol will allow the author to adapt with changes in the academic world, which might increase the rate of acceptance from journal | (Tricco et al., 2008) |
| Type of systematic review | The systematic reviews were divided into: Effectiveness reviews, Experiential (Qualitative) reviews, Costs/Economic Evaluation reviews, Prevalence and/or Incidence reviews, Diagnostic Test Accuracy reviews, Etiology and/or Risk reviews, Expert opinion/policy reviews, Psychometric reviews, Prognostic reviews, Methodological systematic reviews | Different types of systematic reviews might slightly differ in methods, which could lead different obstacles and different chances of publication | (Tricco et al., 2008, Garner et al., 2016) |
| Journal metrics | Impact factor (IF), the number of citations, Citescore, SNIP, and SJR were collected | The journal metrics were described for each database | (Colledge et al., 2010, Da Silva and Memon, 2017) |
| Time from protocol registration to paper publication | Each protocol's publication date was obtained from the corresponding registered database. As long as the actual publishing took place on or before 2013, we picked 2013 as the publication year if it was co-registered. In cases a protocol has more than one publication, the earliest was chosen. From the protocol's publishing date to the paper's publication date, the time from protocol registration to paper publication was calculated | The time from protocol registration to paper publication were described for each database |  |

**References:**

BORAH, R., BROWN, A. W., CAPERS, P. L. & KAISER, K. A. 2017. Analysis of the time and workers needed to conduct systematic reviews of medical interventions using data from the PROSPERO registry. *BMJ Open,* 7**,** e012545.

COLLEDGE, L., DE MOYA-ANEGÓN, F., GUERRERO-BOTE, V. P., LÓPEZ-ILLESCAS, C. & MOED, H. F. 2010. SJR and SNIP: two new journal metrics in Elsevier's Scopus. *Insights,* 23**,** 215.

D'ARRIETTA, L. M., VANGAVETI, V. N., CROWE, M. J. & MALAU-ADULI, B. S. 2022. Rethinking Health Professionals' Motivation to Do Research: A Systematic Review. *J Multidiscip Healthc,* 15**,** 185-216.

DA SILVA, J. A. T. & MEMON, A. R. 2017. CiteScore: A cite for sore eyes, or a valuable, transparent metric? *Scientometrics,* 111**,** 553-556.

DI BITETTI, M. S. & FERRERAS, J. A. 2017. Publish (in English) or perish: The effect on citation rate of using languages other than English in scientific publications. *Ambio,* 46**,** 121-127.

GARNER, P., HOPEWELL, S., CHANDLER, J., MACLEHOSE, H., SCHÜNEMANN, H. J., AKL, E. A., BEYENE, J., CHANG, S., CHURCHILL, R., DEARNESS, K., GUYATT, G., LEFEBVRE, C., LILES, B., MARSHALL, R., MARTÍNEZ GARCÍA, L., MAVERGAMES, C., NASSER, M., QASEEM, A., SAMPSON, M., SOARES-WEISER, K., TAKWOINGI, Y., THABANE, L., TRIVELLA, M., TUGWELL, P., WELSH, E., WILSON, E. C. & SCHÜNEMANN, H. J. 2016. When and how to update systematic reviews: consensus and checklist. *Bmj,* 354**,** i3507.

GÓMEZ-GARCÍA, F., RUANO, J., AGUILAR-LUQUE, M., GAY-MIMBRERA, J., MAESTRE-LOPEZ, B., SANZ-CABANILLAS, J. L., CARMONA-FERNÁNDEZ, P. J., GONZÁLEZ-PADILLA, M., VÉLEZ GARCÍA-NIETO, A. & ISLA-TEJERA, B. 2017. Systematic reviews and meta-analyses on psoriasis: role of funding sources, conflict of interest and bibliometric indices as predictors of methodological quality. *Br J Dermatol,* 176**,** 1633-1644.

HIGGINS, J. & (EDITORS), S. G. 2011. Cochrane handbook for systematic reviews of interventions version 5.1. 0 [updated March 2011]. The Cochrane Collaboration.

PETTICREW, M., WILSON, P., WRIGHT, K. & SONG, F. 2002. Quality of Cochrane reviews. Quality of Cochrane reviews is better than that of non-Cochrane reviews. *Bmj,* 324**,** 545.

SANZ-CABANILLAS, J. L., RUANO, J., GOMEZ-GARCIA, F., ALCALDE-MELLADO, P., GAY-MIMBRERA, J., AGUILAR-LUQUE, M., MAESTRE-LOPEZ, B., GONZALEZ-PADILLA, M., CARMONA-FERNANDEZ, P. J., VELEZ GARCIA-NIETO, A. & ISLA-TEJERA, B. 2017. Author-paper affiliation network architecture influences the methodological quality of systematic reviews and meta-analyses of psoriasis. *PLoS One,* 12**,** e0175419.

SCHREIBER, W. E. & GIUSTINI, D. M. 2019. Measuring Scientific Impact With the h-Index: A Primer for Pathologists. *Am J Clin Pathol,* 151**,** 286-291.

SIDERI, S., PAPAGEORGIOU, S. N. & ELIADES, T. 2018. Registration in the international prospective register of systematic reviews (PROSPERO) of systematic review protocols was associated with increased review quality. *J Clin Epidemiol,* 100**,** 103-110.

TRICCO, A. C., BREHAUT, J., CHEN, M. H. & MOHER, D. 2008. Following 411 Cochrane protocols to completion: a retrospective cohort study. *PLoS One,* 3**,** e3684.

YOUSEFI-NOORAIE, R., SHAKIBA, B. & MORTAZ-HEJRI, S. 2006. Country development and manuscript selection bias: a review of published studies. *BMC Med Res Methodol,* 6**,** 37.
